# Is predictability salient? A study of attentional capture by auditory patterns

**DOI:** 10.1098/rstb.2016.0105

**Published:** 2017-02-19

**Authors:** Rosy Southwell, Anna Baumann, Cécile Gal, Nicolas Barascud, Karl Friston, Maria Chait

**Affiliations:** 1 Ear Institute, University College London, London WC1X 8EE, UK; 2 École Normale Supérieure, Paris 75005, France; 3 Wellcome Trust Centre for Neuroimaging, University College London, London WC1N 3BG, UK

**Keywords:** predictive coding, electroencephalography, attention, statistical learning, regularity, auditory scene analysis

## Abstract

In this series of behavioural and electroencephalography (EEG) experiments, we investigate the extent to which repeating patterns of sounds capture attention. Work in the visual domain has revealed attentional capture by statistically predictable stimuli, consistent with predictive coding accounts which suggest that attention is drawn to sensory regularities. Here, stimuli comprised rapid sequences of tone pips, arranged in regular (REG) or random (RAND) patterns. EEG data demonstrate that the brain rapidly recognizes predictable patterns manifested as a rapid increase in responses to REG relative to RAND sequences. This increase is reminiscent of the increase in gain on neural responses to attended stimuli often seen in the neuroimaging literature, and thus consistent with the hypothesis that predictable sequences draw attention. To study potential attentional capture by auditory regularities, we used REG and RAND sequences in two different behavioural tasks designed to reveal effects of attentional capture by regularity. Overall, the pattern of results suggests that regularity does not capture attention.

This article is part of the themed issue ‘Auditory and visual scene analysis’.

## 1. Introduction

The human brain is highly sensitive to patterns in sensory input [1–5]. A growing body of work in vision [3,6], touch [7], language [1] and audition [8–13] has demonstrated that subjects rapidly and automatically learn complex sensory statistics, and that these are exploited to improve perceptual inference, even when outside conscious awareness. This capacity is often interpreted as a fundamental element of the predictive mechanisms, which are proposed to constitute the principal substrate of perception [14–16].

In hearing, automatic sequence learning has commonly been studied via the mismatch negativity (MMN), an electrophysiological marker for the processing of sounds that break an established rule [11]. MMN to sequence violations has provided (indirect) evidence that the auditory system can learn complex rules governing sequences [10,17]. The repetition positivity, which increases with the number of repeated stimuli, is another neural marker of simple regularity extraction [18]. Recently, Barascud *et al*. [19] provided direct evidence of the process of regularity extraction in more complex tone sequences. They used rapid sequences of tones with frequencies changing in a regular, cyclical pattern, and matched sequences of tones arranged in a random order. Behavioural reaction times (RT) and neural response dynamics indicated rapid recognition of regularity, on par with the latency predicted from an ideal observer model.

Learned knowledge about regularities, whether from low-level statistical learning or conceptual understanding of the phenomena causing sounds, enables predictions to be formed about future sensory input [20]. Such expectations improve behavioural performance in predictable contexts; for example, by orienting resources to a point in time when a stimulus is expected [21], or by facilitating selective attention and segregation of concurrent sound streams [10,22–24]. In addition, recognition of regularities can aid detection of changes in the environment, which causes sensory input that is in disagreement with these predictions [9,25,26]. It has been proposed that the same predictive mechanisms underlie both the detection of regularity violations and auditory scene analysis [20,25].

Attention allows the prioritization of useful streams of information for further processing. Within this context, the relationship between predictability and attention is attracting increasing research interest [27–31]. However, there is disagreement as to whether it is unpredictable, surprising events [32–34] or predictable ones [35] that are the most informative in scene analysis, and should therefore (in the parlance of Itti & Koch [36,37]) be flagged as more ‘salient’ and attract selective attention (see also [38–40]). In visual studies, it has been shown that learning of regularities helps guide attention to expected locations [41,42] and features [41,43]. Zhao *et al*. [44] recently proposed a framework for attentional guidance whereby automatically learned regularities in the sensorium bias attention, even if not relevant for performing a task, and demonstrated this to operate in guiding visual search. They presented sequences of abstract shapes; the order of which was statistically structured at a particular location in the search array and random at others. This was followed by a static visual search array. RT were faster to targets presented at the statistically structured array location, despite the regularity carrying no predictive information as to the target location.

Zhao *et al*. [44] suggest that the prioritization of regular features is a means to focus resources on stable aspects of the world, which can then be learnt. The notion that the brain is ‘hardwired’ to prioritize regularities is at the heart of popular models of the brain as a statistical organ of prediction. The expected precision of bottom-up information streams plays a vital role in such predictive processing accounts [14,31]. Reliable prediction errors are up-weighted in proportion to their expected precision, thereby refining the brain's generative model based on the most informative streams [31].

These ideas may help explain an intriguing recent finding concerning the passive brain response to acoustic patterns. Barascud *et al*. [19] found a substantial increase in the neural response to regularly repeating sound sequences over similar random sequences. This finding seems contrary to a large body of work showing reduced responses to predictable stimuli [45–50]. The proposed explanation for the discrepancy is that, unlike many of the signals used in previous work which often consist of oddball or roving sequences [45,51], the stimuli used in [19] were complex auditory patterns where the predictability of sound sequences was not confounded with neural adaptation resulting from repetition of identical sounds. There are several possible explanations for the increased response to regularity. One is that it reflects the engagement of neural circuits for sequence learning, whose activity in addition to the basic response to the stimulus in auditory cortex results in a net increase in magnetic field strength. Another is that the same neural population is simply more active, with the effect resulting from an increased gain on the activity of auditory neurons responding to the stimuli, potentially signalling greater expected precision. At the cognitive level, the result could potentially indicate that subjects were having their attention spontaneously biased towards the regular sounds, even though they were engaging in an unrelated visual task. Indeed, it has been shown that neural response magnitude is enhanced to attended, predictable stimuli in audition [52,53], and in vision [29].

**Figure 1.**
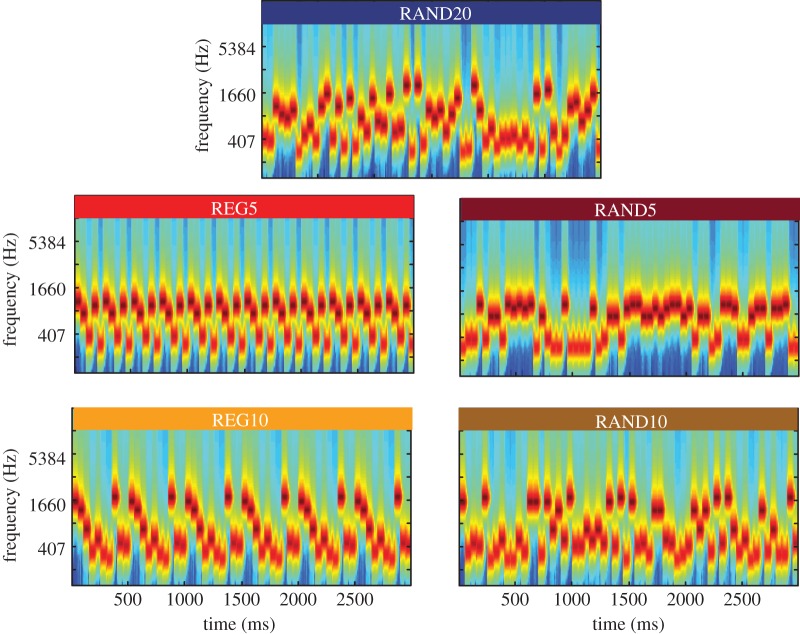
Example spectrograms of the RAND and REG stimuli used. RAND20 (top) contains all 20 frequencies from the pool, in random order. REG5 and REG10 (left) consist of a regularly repeating pattern of 5 or 10 tones, with frequencies chosen at random from the pool (REG15 was also used but not shown). For each REG stimulus, a matching RAND stimulus, consisting of the same frequencies but in random order, was generated. All stimuli were unique (never repeated) and generated anew for each participant.

In the experiments presented in this paper, we investigate whether regularity captures (exogenous) attention. We use the same stimuli as [19], consisting of tone-pip sequences whose frequency pattern is either regularly repeating (REG) or random (RAND; figure 1). In Experiment 1, we demonstrate that the increased brain response to REG relative to RAND also occurs in electroencephalography (EEG). In a series of behavioural experiments, we then investigate the capacity of REG and RAND to exogenously capture attention when they act as auditory distractors (Experiment 2) and test whether auditory regularity biases attention in scenarios where multiple sound streams are attended and task-relevant (Experiment 3). In both of these paradigms, we find no evidence for attentional capture by acoustic regularity.

## 2. Experiment 1 (electroencephalography)

Experiment 1 investigated EEG brain responses to regularly repeating (REG) and random (RAND) tone-pip sequences of varying complexity (figure 1) while participants, naive to the auditory stimuli, were engaged in an unrelated visual task. The stimuli were identical to those previously used by Barascud *et al.* [19] in a magnetoencephalography (MEG) study. The main aim was to replicate these results using EEG. In addition to the differences in sensitivity between the two techniques [54], a replication in EEG is key to assimilating those findings with the existing literature, where the majority of electrophysiology studies on regularity detection use EEG.

### (a) Methods

#### (i) Stimuli

Stimuli (figure 1) were 3000 ms long sequences of 50 ms tone pips (60 tone pips altogether; each ramped on and off with a 5 ms raised cosine ramp). Tone frequencies were drawn from a pool of 20 logarithmically spaced values between 222–2000 Hz. A unique sequence was presented on each trial. Sequences were defined by two parameters: *Rcyc* (alphabet size)—the number of frequencies chosen (at random, with replacement) from the pool, and *regularity* (REG or RAND). In regular (REG) sequences, a sub-pool of *Rcyc* frequencies were chosen from the full pool, and arranged in repeating cycles of length *Rcyc*. Random (RAND) sequences were generated by drawing each tone at random from the sub-pool of *Rcyc* frequencies. REG and RAND sequences of the same *Rcyc* were generated in pairs, using the same sub-pool, such that conditions were matched for the occurrence of each frequency (figure 1). REG conditions used *Rcyc* = 5, 10 and 15; RAND included an additional condition of *Rcyc* = 20 (using the whole frequency pool), yielding 7 conditions (REG5, REG10, REG15, RAND5, RAND10, RAND15 and RAND20). These sequences are too rapid to allow deliberate reasoning of the order of individual tones; nevertheless, the repetitions in REG sequences lead to a strong, ‘pop-out’ percept of a pattern [19]. Examples of the stimuli used are provided as the electronic supplementary material.

#### (ii) Procedure

The procedure was similar to the MEG experiment described in [19]. Subjects were engaged in an incidental visual task and were naive about the nature of the auditory stimuli. Auditory stimuli were presented binaurally with the Psychophysics Toolbox extension in Matlab [55]. In total, subjects heard 700 unique stimuli (100 for each condition). The inter-stimulus interval (ISI) was jittered between 1100 and 1500 ms. The visual task was displayed on a separate computer using Cogent 2000 in Matlab (www.vislab.ucl.ac.uk/cogent.php). The timing was not correlated with that of the auditory stimuli. For each trial, three colour photographs of landscapes were shown for 5 s each, and images faded gradually from one image to the next to minimize visual transients. Subjects were instructed to press a keyboard button if the first and third image within a trial were identical (10% of trials), and to withhold a response otherwise. Inter-trial interval was jittered between 2 and 5 s. The session was split into four consecutive blocks. Feedback (number of hits, misses and false alarms) for the visual task was provided at the end of each block.

#### (iii) Recording and data preprocessing

EEG signals were recorded using a Biosemi system (Biosemi Active Two AD-box ADC-17, Biosemi, Netherlands) with 64 electrodes; at a sampling rate of 2048 Hz. Recording was re-started at each block. Data were analysed with SPM12 (Statistical Parametric Mapping; http://www.fil.ion.ucl.ac.uk/spm/) and Fieldtrip (http://www.fieldtriptoolbox.org/; [56]) toolboxes for Matlab (2015a, MathWorks). All filtering was performed with a two-pass, Butterworth, fifth order filter. Data were low-pass filtered at 110 Hz, downsampled at 256 Hz, high-pass filtered at 0.1 Hz, re-referenced to the average, divided into 5000 ms epochs (with 1000 ms pre stimulus onset and 1000 ms post-offset) and baseline-corrected relative to the pre-onset interval. Outlier epochs were removed, if the average power over all time samples and channels exceeded 2 s.d. from the mean over trials; on average, 76% of epochs were retained. Subsequently, data were low-pass filtered at 30 Hz and de-noising source separation (DSS; [57,58]) was applied to maximize reproducibility across epochs, keeping the first five components and projecting back into sensor space. Finally, data were averaged over epochs for each channel, condition and subject.

#### (iv) Data analysis

For each participant and condition, the root-mean-square (RMS) over channels was calculated at each time sample in the epoch. This was used as a measure of brain activation over time. The distribution of RMS (mean, s.e.) was then estimated for each condition using bootstrap resampling across subjects (1000 iterations; [59]). This was used to calculate the group-level *t*-statistic of the difference between pairs of conditions at each time-point. *T*-tests (two-tail) were performed using *t*-statistics computed on clusters in time, and controlled for a family-wise error rate of 0.05 [60]. Additionally, a repeated-measures ANOVA with factors of regularity and alphabet size was performed on the mean RMS power between 1000 and 3000 ms, including all conditions except RAND20 to give a balanced design.

#### (v) Subjects

In total, 23 paid subjects took part (mean age 23.3 years, range 20–29 years; 11 female). Two subjects were excluded due to exceptionally noisy EEG data. None reported a history of hearing impairment or neurological disorder.

### (b) Results and discussion

**Figure 2.**
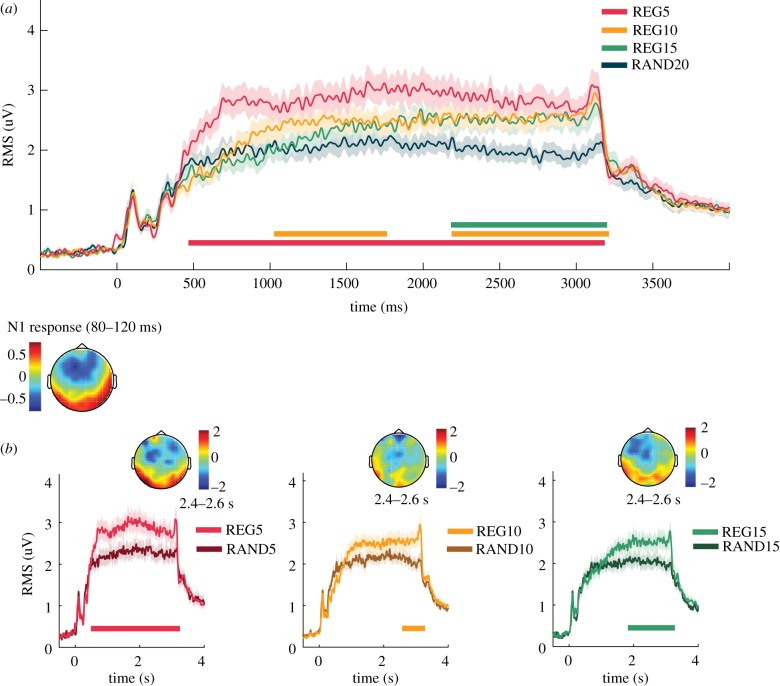
Group results of Experiment 1 (EEG). Shaded error margins show ±2 s.e.m. (*a*) EEG evoked responses (group RMS over all channels) for RAND20 and REG of different *Rcyc*, over the entire epoch. Horizontal bars below plots indicate time intervals where cluster-level statistics showed a significant difference between each of the REG conditions and RAND20. (*b*) The responses to REG are re-plotted alongside their respective RAND controls. Horizontal bars below plots indicate time intervals where cluster-level statistics showed a significant difference between each pair of conditions. The scalp voltage map of the difference between each pair of REG and RAND conditions*,* calculated between 2.4 and 2.6 s post-onset, are plotted above each trace. For comparison to a standard scalp voltage distribution in response to a simple sound, the scalp topography of the N1 (calculated over a 40 ms window centred on 100 ms following stimulus onset) is also provided.

Group RMS (RMS of all subjects' RMSs) for the three regular conditions, REG5, REG10 and REG15, alongside RAND20 as a common control, are shown in figure 2*a*. The brain response shows an N1 peak (at 100 ms post-onset) before rising gradually and reaching a sustained level, which persists until stimulus offset. An offset response is visible about 100 ms after sequence cessation. The sustained evoked response is characterized by regular fluctuations at 20 Hz reflecting responses to individual tones. All three REG conditions show an increased sustained response when compared with RAND20. The timing at which the group RMS for REG conditions diverge from RAND20 (taken to reflect the time required by the brain to discover the regularity) increases with cycle length: 406 ms (8 tones), 750 ms (15 tones), 1067 ms (21 tones); for REG with *Rcyc* = 5, 10, 15, respectively. This is during the second cycle in each case (1.6, 1.5 and 1.4 cycles, respectively), before the pattern has repeated completely, although stable statistical significance is reached somewhat later (horizontal lines beneath the RMS plot). As discussed in [19], this demonstrates the operation of a rapid, automatic process of regularity detection. Group RMS for REG and RAND of matched *Rcyc* are shown in figure 2*b*. The response to REG is consistently higher than its matched RAND. The scalp voltage map of the difference between REG and matched RAND conditions*,* calculated between 2.4 and 2.6 s post-onset, is shown in figure 2*b*. For comparison to a standard scalp voltage distribution in response to sound, the scalp topography of the N1 onset response (calculated over a 40 ms window centred on 100 ms following stimulus onset) is also provided.

The RMS, over the interval 1000–3000 ms post-onset, extracted from each condition, was subjected to a repeated-measures ANOVA with regularity (REG versus RAND) and *Rcyc* (alphabet size of 5, 10 or 15 tones) as within subject factors. This yielded significant main effects of regularity (*F* = 23.2, *p*= <0.001) and of *Rcyc* (*F* = 5.4, *p* = 0.014), with no interactions. The relative mean increase between RAND and REG of matched *Rcyc* was 29.7%, 36.7% and 27.5% for *Rcyc* = 5, 10 and 15, respectively.

Experiment 1 demonstrated a rapid differentiation in brain response between regular and random sequences, replicating the MEG results [19]. However, the EEG responses are somewhat noisier than those measured with MEG, probably influenced by a number of factors including ambient electric noise and the smaller number of sensors used here (64, versus 274 in MEG), which impairs the efficiency of de-noising. Electrode voltage drifts, which introduce low frequency noise, may also have affected the robustness of the observed sustained responses.

Overall, the data demonstrate that regular, predictable sequences lead to a dramatic power increase of some 30%, which is remarkable for evoked responses, and suggests a large change in the underlying neural activity. This finding is surprising in the light of previous work consistently reporting reduced evoked responses to predictable patterns and interpreted as reflecting reduced prediction error (e.g. [47]; for a review see [51]). The discrepancy with previous work may be due to much of the existing work using sound patterns, which confound repetition with predictability, making it difficult to dissociate adaptation effects from those purely due to prediction. The present paradigm, using wide-band signals and complex sound patterns allows us to control for simple effects of adaptation (see additional discussion in [19]). Furthermore, we use very rapid sequences, where the perception of patterns pops-out spontaneously rather than being consciously trackable. It is likely that the neural processes involved in extracting the regularity are different from those implicated in work using slower temporal patterns (e.g. [52,53]), which allow high-level (conscious or mnemonic) prediction of future events.

One possible explanation for the activation pattern observed here is that it reflects automatic, bottom-up–driven attentional capture by REG patterns. This attentional process will be the focus of the rest of this paper. The behavioural experiments below investigate the hypothesis that the large, sustained amplitude shift that was observed for the REG stimuli may reflect increased perceptual salience [36,37]. That is to say, the more reliable REG stimuli trigger an automatic (exogenous) attentional bias. This proposition leads to the testable prediction that REG and RAND will have different effects on behaviour, reflecting an attentional bias towards regularity in REG sequences, even when task-irrelevant.

## 3. Experiment 2

This experiment aimed to measure the (assumed) behavioural consequences of attentional capture by regular sounds. We evaluated performance on a demanding listening task, with REG or RAND sequences presented concurrently, as task-irrelevant distractors. If REG patterns spontaneously capture exogenous attention, we predicted that REG sequences will prove more detrimental to performance than RAND sequences. This prediction is in line with previous behavioural experiments, whereby task-irrelevant stimuli outside the focus of attention can result in attentional capture manifest as degradation in performance in a behavioural task [61,62].

The main task was based on an auditory change-detection paradigm [63,64]. Stimuli were artificial acoustic scenes, comprised of multiple simultaneous tone-pip streams, each characterized by a distinct, constant frequency and amplitude-modulation (AM) rate. The task required listeners to detect occasional changes (appearance or disappearance of one stream) within these scenes. This simulates the challenges faced by listeners in natural acoustic scenes, in which many concurrent sound sources must be processed and monitored simultaneously.

**Figure 3.**
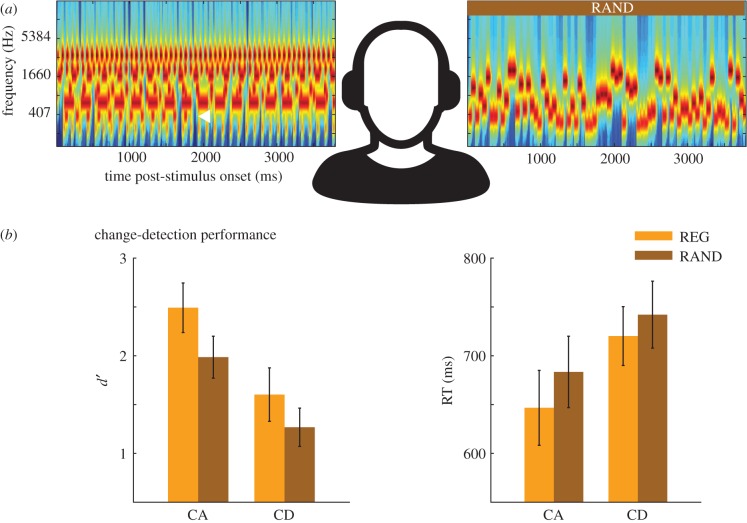
Experiment 2. (*a*) A schematic representation of the stimulus paradigm. Scene stimuli and REG or RAND sequences (RAND in this example) were presented concurrently to different ears (counterbalanced across subjects). All scenes contained eight streams. This example shows a scene with a disappearing (CD) stream, which is indicated with a white arrowhead. (*b*) Behavioural results; *d*’ (left) and reaction time (right) for detection of change events. Error bars show ±1 s.e.m.

We presented the change-detection task and REG-RAND distractor sequences concurrently to different ears, such that they competed directly throughout the trial (figure 3*a*). If REG patterns spontaneously capture exogenous attention, we predicted that REG sequences will prove more detrimental to performance than RAND sequences.

### (a) Methods

#### (i) Stimuli

The stimuli and experimental approach for the change-detection paradigm are described in detail in a previous study [65]. In brief, stimuli were artificial acoustic scenes consisting of eight concurrent streams of tone pips, each with a unique frequency (between 200 and 4000 Hz) and AM rate (3 to 35 Hz). In total, 50% of the stimuli contained a change partway through the scene: appearance (CA) or disappearance (CD) of a stream. Scene changes occurred between 1000 and 2000 ms post-onset. The overall stimulus duration was between 2000 and 4000 ms.

On each trial, a scene stimulus and a REG10 or RAND10 distractor sequence (with equal probability) were presented concurrently at the same dB level, to different ears, such that they competed directly throughout the trial (figure 3*a*). Each REG-RAND sequence consisted of between 40 and 80 tones. For REG sequences, this constituted between 4 and 8 cycles; i.e. sufficient for the regularity to become perceptually established.

#### (ii) Procedure

Stimuli were blocked by change type (CA or CD), with 50% of the trials in each block (160 overall) containing a change. The ISI was randomized between 700 and 2000 ms. REG or RAND sequences were randomly paired with each scene stimulus. Subjects were instructed to attend to the ear containing the scene and respond by button-press as soon as they heard a change. To avoid confusion, the ear of presentation was fixed throughout the experiment, but counterbalanced across subjects. Subjects were naive to the structure of the REG-RAND sequences, and told these were simply distractors to the main change-detection task. The block order was counterbalanced between subjects, and a break was allowed after every 40 trials. The session began with a short training block where feedback was given on each trial.

#### (iii) Subjects

Ten subjects participated in this experiment (mean age 24.0 years; 7 female).

### (b) Results and discussion

Figure 3*b* shows RT and sensitivity (*d*’) scores for detection of scene changes with REG or RAND distractors presented concurrently. A repeated-measures ANOVA was performed on RT and *d*’; with change type (CA/CD) and distractor regularity (REG/RAND) as factors. RT showed main effects of change type (*F* = 16.299; *p* = 0.003) and regularity (*F* = 8.064; *p* = 0.019) with no interaction. Similarly, *d*’ showed a main effect of change type (*F* = 18.244; *p* = 0.002) and regularity (*F* = 9.786; *p* = 0.012) with no interaction.

The results reveal that, contrary to our hypothesis, RAND is more detrimental to performance than REG. The data are consistent with the interpretation that RAND is harder to ignore, at least when in direct competition with a concurrent, task-relevant, auditory stream (see also [23]; more discussion below).

## 4. Experiment 3

Rather than using REG or RAND sequences as task-irrelevant distractors, Experiment 3 placed REG and RAND sequences in direct competition as task-relevant streams. Here, REG and RAND were presented concurrently and both actively monitored for targets (a silent gap). This is in contrast with Experiment 2, where performance on the task required ignoring REG or RAND stimuli. We predicted that when both sequence types are monitored simultaneously, gaps in REG sequences should be more readily detectable than gaps in RAND sequences. This design is similar to that used in [44], who demonstrated that targets embedded within regularly repeating visual streams are more easily detected, even though the regularity of the stream was not itself goal-relevant.

### (a) Methods

#### (i) Stimuli

**Figure 4.**
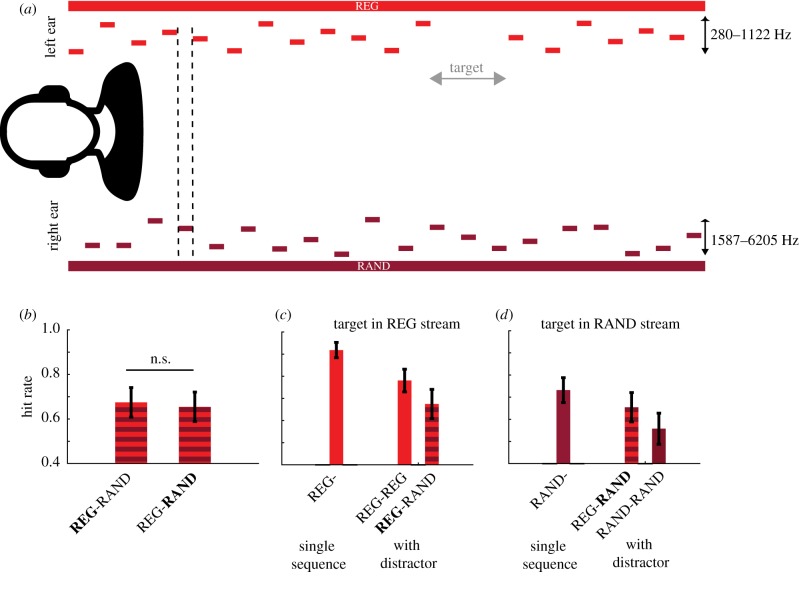
Experiment 3. (*a*) A schematic representation of the stimulus paradigm. On each trial, two concurrent sequences (each either REG or RAND) were presented, one to each ear. Individual tones were interleaved as demonstrated with the black dashed line. On 50% of the trials, one of the sequences contained a 200 ms gap (target, shown in grey, top). The sequences presented to the right ear were of a higher pitch than to the left, such that the frequency ranges did not overlap. (*b*) Hit rate for target detection when REG was presented to one ear and RAND to the other. The sequence containing the target is indicated in bold. (*c*) Hit rate for all conditions with a target in the REG stream; single-sequence REG (left) and dual-sequence (right). (*d*) As for C; except all conditions had a target in the RAND stream. Error bars show ±1 s.e.m.

This experiment used REG5 and RAND5 sequences consisting of 50 ms tone pips interspersed with 50 ms gaps. Trials involved the presentation of two concurrent sequences, one in each ear (figure 4*a*). Sequences could be both REG or both RAND, or one of each. In order to facilitate the perception of the two sequences as independent concurrent streams, the sequences were staggered by 50 ms, such that tones occurred in alternation between the ears. In addition, sequences were spectrally separated, such that the sequence in the right ear was always a higher pitch. The tones were chosen from a pool of 13 logarithmically spaced frequencies between 1587 and 6205 Hz for the right ear and between 280 and 1122 Hz for the left.

On 50% of trials, one of the sequences contained a target (an omission of two consecutive tones). Stimuli were 6000 ms long. When present, the target occurred at least 2000 ms after stimulus onset (following four REG cycles); i.e. at a point in the REG stimulus when the regular pattern has been established.

The main experiment consisted of the following conditions: (i) RAND sequences in both ears (RAND-RAND, 25% of trials); (ii) REG sequences in both ears (REG-REG, 25% of trials); (iii) REG sequence in one ear and RAND in the other (REG-RAND, 50% of trials). The target occurred in one of the two sequences with equal probability. For REG-RAND, we denote the stream containing the target using bold type; thereby sub-dividing this condition into (iii(a)) **REG**-RAND and (iii(b)) REG-**RAND**. Each condition was counterbalanced across the two ears, such that target occurrence and REG versus RAND sequences were equally likely in each ear. Before the main session, subjects also completed a block where only a single-sequence (REG or RAND) was presented to one of the ears with equal probability; a target was present on 50% of trials. These conditions are denoted as REG- and RAND-.

#### (ii) Procedure

Subjects were instructed to press a keyboard button as soon as they heard a gap in a sequence. Feedback was provided after each trial. The single-sequence block contained 80 stimuli (40 for each of REG- and RAND-). The main experiment contained 120 trials for each of the four dual-sequence conditions, presented in a randomized order. Subjects were given short breaks every 10 min.

#### (iii) Subjects

Eleven subjects participated in this experiment (mean age = 25.7; 7 female)

### (b) Results and discussion

We used hit rates as our primary outcome measure, as most subjects produced low false positive rates (2.1% for single-sequence, 2.0% for dual-sequence) yielding ‘artificially’ high *d*’ scores. Hit rates were deemed the most unambiguous and representative outcome measure of performance in this task.

In order to test the main hypothesis that regular sequences would ‘pop-out’ and attract attention, we initially compared performance for **REG**-RAND and REG-**RAND**, as both contain simultaneously presented random and regular sequences (figure 4*b*). We postulated that regularity would bias attention, leading to improved performance when targets were embedded in regular streams (**REG**-RAND) as opposed to random (REG-**RAND)**. A repeated-measures ANOVA showed no significant difference between the average hit rate values for **REG**-RAND and REG-**RAND** (hit rates were 0.65 and 0.67, respectively; *F* = 1.2; *p* = 0.3). These results suggest that regular sound patterns do not bias attention.

Figure 4*c*,*d* shows the hit rate for all conditions, separated by whether the target is in the REG or the RAND stream—for both single-sequence and dual-sequence stimuli. The hit rates for targets in the single-sequence (REG- and RAND-) conditions are the left-most bars in each plot. In the single-sequence condition, subjects were better at identifying targets in regular streams compared with random streams (*F* = 20.6; *p* = 0.001). These findings are consistent with previous work showing that performance is improved when targets are embedded in temporally regular sequences [66–68]. This is the case even when the dimension in which the regularity expressed is independent of the dimension along which targets differ.

A repeated-measures ANOVA was conducted on hit rates in the dual-sequence conditions, with factors for regularity of the target stream (REG-RAND) and the parallel stream (REG-RAND). There was a main effect of target stream (*F* = 51.48; *p* < 0.01); here again subjects were overall better at detecting targets in REG. We also found a main effect of parallel stream (*F* = 26.97; *p* < 0.01); revealing overall poorer target detection when the parallel stream was RAND relative to when it was REG. This pattern is in agreement with the outcomes of Experiment 2, and consistent with the interpretation that RAND patterns incur increased demand on processing resources (discussed further below). However, there was no interaction between the two factors, suggesting that a RAND parallel stream was equally costly to target-detection performance in a RAND or a REG stream.

## 5. Discussion

Brain responses measured with functional magnetic resonance imaging (fMRI), MEG [19] and EEG (as seen here) show consistently increased activation to regular acoustic patterns, relative to matched random stimuli. One interpretation of these systematic, pronounced effects is that they indicate large differences in attentional capture between regular and random patterns, such that regular patterns automatically and involuntarily attract more attention. This account of the imaging data is consistent with previous behavioural work in the visual modality [44] and is broadly in line with the fact that sensitivity to predictable patterns in the natural environment is a major pre-requisite for survival. Organisms produce regular, periodic motor sequences, such as locomotion and vocalizations, which are expressed as a pattern in the temporal succession of sensations. The ability to automatically orient towards such patterns within a crowded, noisy scene is often critical for continued existence.

### (a) Attentional capture by regularity?

The behavioural experiments reported here aimed to identify a behavioural correlate for the observed brain response effects. Using two tasks designed to probe different aspects of attentional capture, we consistently find no evidence for the exogenous capture of attention by regular acoustic patterns. Despite the sizeable change in the EEG signal associated with processing regular (REG), relative to random (RAND) tone-pip patterns, REG sequences were not more distracting than matched random sequences when task irrelevant, and were also no more perceptually salient when participants were actively monitoring REG and RAND streams concurrently.

While there is no evidence for REG sequences being more perceptually salient than RAND sequences (or vice versa), Experiments 2 and 3 suggest that RAND sequences are more computationally demanding, and hence more distracting, than REG sequences (e.g. [69]; the discussion below disentangles these issues).

The paradigm in Experiment 2 shared key similarities with the EEG experiment (Experiment 1). Participants were naive to the nature of the distracting REG or RAND patterns, and focused on a different task. A change-detection task, rather than a visual task similar to that in the EEG experiment, was chosen because: (i) its rapid nature allowed us to probe behaviour more frequently, and hence efficiently; and (ii) a competing auditory (rather than a visual) task is more likely to reveal effects of distraction, because it poses more competition for shared resources (see review in [70,71]). It is therefore unlikely that failure to observe effects of attentional capture is due to the difference in task *per se*. Furthermore, by removing the decoy task altogether, Experiment 3 constitutes a stricter test for a possible attentional bias. When REG and RAND are monitored concurrently, we observe equal gap-detection performance whether the target is in REG or RAND, which suggests that they do not differ in their perceptual salience.

The reasons for the discrepancy with results from vision [44], where effects of attentional capture by regularity have been reported, are unclear and may be due to many factors, perhaps including a genuine difference in the mechanisms of attentional allocation in the visual and auditory domain. Further work directly comparing the two modalities is required to resolve this issue.

### (b) Processing of regular versus random sequences

The results of Experiment 3, demonstrating increased sensitivity to targets in REG relative to RAND sequences when presented alone, are consistent with many previous demonstrations that regularity facilitates behavioural performance. These studies, albeit mostly using regularity in the temporal dimension rather than in frequency as we do here, consistently show that regularity facilitates behavioural performance. Expected events are detected and assessed more rapidly and accurately than unexpected events [61–64,66,67,72–75]. This occurs, as is the case here, even when the task dimension is orthogonal to the feature dimension over which the regularity is defined (e.g. [68]) and hypothesized to arise due to the ‘pre-activation’ of the relevant neural machinery for processing-predicted events [63,76].

The same processes have been demonstrated to contribute to the *suppression* of regular streams when they are not behaviourally relevant. For example, Andreou *et al.* [23] demonstrated that it is easier to ignore a temporally regular sequence, relative to a temporally irregular sequence (see also [24,77,78]). Similarly, in Experiment 2, we show that REG sequences are less distracting than RAND sequences when participants are required to ignore those sequences and focus on a competing change-detection task. A potential mechanism for this effect is supplied by predictive coding [15,16], whereby predictable inputs are attenuated by top-down predictions, and the resulting prediction error triggers a process of updating the internal predictive model. Regularity allows the derivation of a predictive rule; therefore, it becomes easier to ‘explain away’ the irrelevant stimulus, by suppressing the prediction error with a closely matching top-down prediction. Irregular stimuli demand more resources for processing as they elicit a constant stream of prediction errors and thus constantly trigger model updating. This may be taken to suggest that (unpredictable) RAND sequences are more perceptually salient. From the point of view of predictive coding, this is sensible because RAND sequences are characterized by higher information content than REG sequences. Friston *et al.* [79] define salience in terms of the ability to reduce uncertainty or to inform hypotheses about the sensory scene being sampled. In the visual domain, this is usually measured in terms of Bayesian surprise [33] and more generally as information gain or epistemic value [80]. However, whether the theoretically information-rich RAND signals are useful in reducing uncertainty about high-level representations is an open question. In other words, is unpredictability itself salient? The lack of a bias in performance between REG and RAND sequences presented in direct competition (Experiment 3) suggests not.

### (c) If not attentional capture, what is the source of the electroencephalography effect?

EEG and MEG measurements demonstrate an increase in the amplitude of the sustained response for REG relative to RAND stimuli. In fMRI, this is associated with greater activation for REG relative to RAND sequences across a large portion of the superior temporal gyrus, including Heschl's gyrus and planum temporale [19]. The behavioural results reported above suggest that this increased activation is not associated with attentional bias towards (or increased perceptual salience of) REG sequences.

Critically, the above explanation for why RAND sequences were, in some cases, more detrimental to performance than REG sequences implies *more* activation (increased demand for computational resources) for random patterns relative to regular ones. This may seem contradictory to the brain-level effects. However, it is possible, that the increased auditory cortical activation for regular patterns observed in M/EEG and fMRI reflects increased *inhibition.* It is difficult to dissociate excitatory and inhibitory activation with standard non-invasive brain imaging techniques; rather future computational and electrophysiological tools would be critical for exploring this possibility. Indeed, recent findings in animal models demonstrate a critical role for inhibition in shaping the response of primary auditory cortex neurons to regularly repeating sounds in the context of an oddball paradigm [81].

Another potential explanation for the larger response to REG is that regularity detection is associated with heightened sensitivity (increased gain) of the sensory units activated by the regular pattern. According to this ‘precision-weighting’ account, precise, i.e. highly predictable, sensory streams are preferentially weighted by increasing the post-synaptic gain of the relevant (prediction error) units [14]. Importantly, this can occur within the remit of automatic processing, so does not entail attentional capture [31].

Lastly, it is possible that the increased sustained response we observe is due to another process (or indeed multiple processes) such as learning, working memory or recognition of a match to a memory of previous stimulation (see [19] for further discussion). This interpretation is consistent with the diffuse source network including auditory cortex, hippocampus and inferior frontal gyrus identified in [19] as contributing to the brain response to structured sequences.

To summarize, a picture emerges from these results in which regularity in non-attended items does not capture attention. In fact, as demonstrated in Experiment 2, random stimuli can be more distracting than regular ones. Consistent with the literature, we found that regularity does, however, aid in scene analysis by being easier to ignore (Experiment 2) and requiring fewer resources to process (Experiment 3). Collectively, the behavioural and brain imaging findings can be reconciled by considering both to result from mechanisms that minimize surprise and uncertainty about the world [10,25].

## Supplementary Material

Supplementary auditory material

## Data accessibility

The behavioural and EEG data are available online, alongside Matlab code for analysis and plotting. This is available through the Dryad repository http://doi.org/10.5061/dryad.j17h3 [82].
