# Supplementary material for: Is predictability salient? A study of attentional capture by auditory patterns
Source: Philos Trans R Soc Lond B Biol Sci. 2017 Feb 19;372(1714):20160105. doi: 10.1098/rstb.2016.0105 (PMC5206273; doi:10.1098/rstb.2016.0105)
Supplement: Supplementary auditory material [file rstb20160105supp1.rtf]

Is predictability salient? A study of attentional capture by auditory patterns
Rosy Southwella,*, Anna Baumanna,*, Cécile Gala,*, Nicolas Barascudb, Karl Fristonc &
Maria Chaita
aEar Institute, University College London, London WC1X 8EE, United Kingdom
bÉcole Normale Supérieure, Paris 75005, France
cWellcome Trust Centre for Neuroimaging, University College London, London WC1N 3BG, United Kingdom
*These authors contributed equally to this work.


Supplementary Audio Materials
Included are examples of the REG and RAND sounds used in Experiment 1 and 2 (2 instances for each condition, see filenames). 

REG5
REG10
REG15
RAND5
RAND10
RAND15
RAND20
